# Soft Wireless Headband Bioelectronics and Electrooculography for Persistent Human–Machine Interfaces

**DOI:** 10.1021/acsaelm.2c01436

**Published:** 2023-02-08

**Authors:** Seunghyeb Ban, Yoon Jae Lee, Shinjae Kwon, Yun-Soung Kim, Jae Won Chang, Jong-Hoon Kim, Woon-Hong Yeo

**Affiliations:** † School of Engineering and Computer Science, Washington State University, Vancouver, Washington 98686, United States; ‡ IEN Center for Human-Centric Interfaces and Engineering at the Institute for Electronics and Nanotechnology, Georgia Institute of Technology, Atlanta, Georgia 30332, United States; § School of Electrical and Computer Engineering, Georgia Institute of Technology, Atlanta, Georgia 30332, United States; ∥ George W. Woodruff School of Mechanical Engineering, College of Engineering, Georgia Institute of Technology, Atlanta, Georgia 30332, United States; ⊥ BioMedical Engineering and Imaging Institute, Icahn School of Medicine at Mount Sinai, New York, New York 10029, United States; # Department of Otolaryngology Head and Neck Surgery, School of Medicine, Chungnam National University Hospital, Daejeon 35015, Republic of Korea; ¶ Department of Mechanical Engineering, University of Washington, Seattle, Washington 98195, United States; ∇ Wallace H. Coulter Department of Biomedical Engineering, Georgia Institute of Technology and Emory University School of Medicine, Atlanta, Georgia 30332, United States; ○ Parker H. Petit Institute for Bioengineering and Biosciences, Institute for Materials, Neural Engineering Center, Institute for Robotics and Intelligent Machines, Georgia Institute of Technology, Atlanta, Georgia 30332, United States

**Keywords:** soft materials, flexible headband, wireless bioelectronics, electrooculography, deep learning, real-time classification, human−machine interface

## Abstract

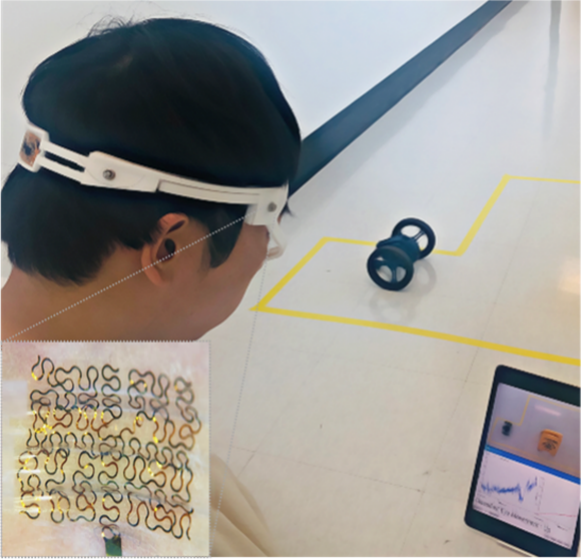

Recent advances in wearable technologies have enabled ways for people to interact with external devices, known as human–machine interfaces (HMIs). Among them, electrooculography (EOG), measured by wearable devices, is used for eye movement-enabled HMI. Most prior studies have utilized conventional gel electrodes for EOG recording. However, the gel is problematic due to skin irritation, while separate bulky electronics cause motion artifacts. Here, we introduce a low-profile, headband-type, soft wearable electronic system with embedded stretchable electrodes, and a flexible wireless circuit to detect EOG signals for persistent HMIs. The headband with dry electrodes is printed with flexible thermoplastic polyurethane. Nanomembrane electrodes are prepared by thin-film deposition and laser cutting techniques. A set of signal processing data from dry electrodes demonstrate successful real-time classification of eye motions, including blink, up, down, left, and right. Our study shows that the convolutional neural network performs exceptionally well compared to other machine learning methods, showing 98.3% accuracy with six classes: the highest performance till date in EOG classification with only four electrodes. Collectively, the real-time demonstration of continuous wireless control of a two-wheeled radio-controlled car captures the potential of the bioelectronic system and the algorithm for targeting various HMI and virtual reality applications.

## Introduction

The need for human–machine interface (HMI) technology connecting healthcare applications is increasing rapidly. For example, a touch screen and joystick are HMI, a user interface connecting a person to a machine. The global HMI market is expected to generate more than $8 billion in revenue from 2017 to 2023.^1^ Among various HMI fields, the field of healthcare is receiving a lot of attention. Recent studies have shown that the wheelchair based on HMI was developed to aid disabled people in their daily activities.^2−5^ Typical input signals for HMI are body motions such as hand or finger motion and biopotential. Healthcare applications require an ergonomic approach and high precision.^6^ In this case, biopotential signals are attractive candidates since biopotential is non-invasive, requires minimal hardware, and contains user movement information. That is why physiological biopotentials and human activities that wearable devices can measure have been suggested for HMI, such as controllers for various healthcare applications. Physiological biopotentials, such as electromyography (EMG), electroencephalography (EEG), and electrooculography (EOG), can be the control commands. For example, EMG signals from muscle movements with a fast response have proved possible to connect with HMI.^7^ However, muscle weakness due to disabilities cannot produce the required stimulus for the detection of EMG.^8^ EEG can be another way, which exploits neural information as input control for HMI. However, prior studies further indicated that noninvasive EEG features do not contain sufficient information about small movements.^9^ High-fidelity EEG is also difficult to acquire and not feasible for real-time and accurate HMI applications. When measured from the scalp, an EEG signal has an amplitude between about 10 and 100 μV. However, EOG amplitude, which has an amplitude between about 0.05 and 3 mV, is quite larger than EEG amplitude.^10−13^ Due to frail grip strength and issues with controlling their bodies for existing motorized wheelchair users,^14−18^ there are restrictions on the use of EMG and EEG. As one of the technologies for tracking eye movements by measuring the potential via the positively charged cornea and negatively charged retina, EOG has gained interest in HMI.^19^

In several papers, wearable EOG devices in the form of glasses have been used because of their easy and fast wear. For example, the conventional wearable EOG eyeglasses named JINS MEME have one electrode on the bridge of the nose and one on each of the nose pads of the eyeglasses.^5,20^ Some studies also manufactured 3D printed glasses-type wearable EOG devices.^21^ However, a glasses-type device is inconvenient to people who are already wearing glasses. These devices are restricted when the electrode is secured to the skin or when there is movement. In addition, glasses-type platforms can be challenging to wear for people with a variety of head sizes because glasses-type platforms are made with a fixed frame width and temple length. Since the glasses-type platform was not size-adjustable, it should be used by users who fit the prescribed size of glasses. Also, wearing it in an inappropriate head size can cause the glasses-type platform to come off during active movements. From the perspective of electrodes, prior studies using the gel electrode show high-fidelity recording, the existing electrodes still have limitations, such as poor breathability, skin irritation, and loss of performance during long-term monitoring due to drying. The conventional gel electrodes dehydrate and reduce the electrode performance over time.^22^ For the aforementioned reasons, the gel electrodes should be changed periodically. Constant changing of electrodes is not convenient in healthcare applications and is inefficient.^23^ On the other hand, prior work demonstrated HMI using eye-tracking capability within wearable devices by integrating infrared cameras.^24^ This HMI using eye-tracking has several problems. There should always be a camera that blocks that person’s view. This system also requires clear pupil and eye images of the user. Still, eyelashes and eyelids can hinder the successful detection of the pupil and bright light can also interfere with pupil detection.^25^

In this work, we introduce a soft material-based, all-in-one headband EOG device integrating a flexible wireless circuit and an array of fractal gold electrodes that compensate for the limitations of the pre-existing devices mentioned above. The headband platform is advantageous over glasses-type counterparts such as a size-adjustable and stable adhesion. In the case of a glasses-type platform, the part that supports the face is narrow, but the headband type platform has a wider electrode-skin contact area, so multiple electrodes can be secured to the face. To address the gel issues such as skin irritation and short-term durability, we introduce ultrathin, dry electrodes. In recent papers, with its well-studied biocompatibility and processibility, mesh-patterned gold electrodes have been widely used to measure biopotentials.^26,27^ Specifically, the ultrathin, fractal-designed gold electrode can help the electrode accommodate dynamic skin deformation for a high-fidelity recording of EOG and causes fewer skin irritations compared to the existing gel electrodes. Also, the wearable EOG device can acquire EOG data and classify eye directions in real-time. Compared to prior articles within the scope of real-time classification of eye movements based on wearable EOG devices, our device shows the highest accuracy in classifying six different classes with only four electrodes. Overall, the presented system can meet requirements such as an ergonomic approach and high-precision interfaces. The wearable EOG device with this system allows people to acquire EOG signals stably and control various healthcare applications.

## Results and Discussion

### Overview of a Wireless, Portable Wearable EOG Device

**Figure 1:**
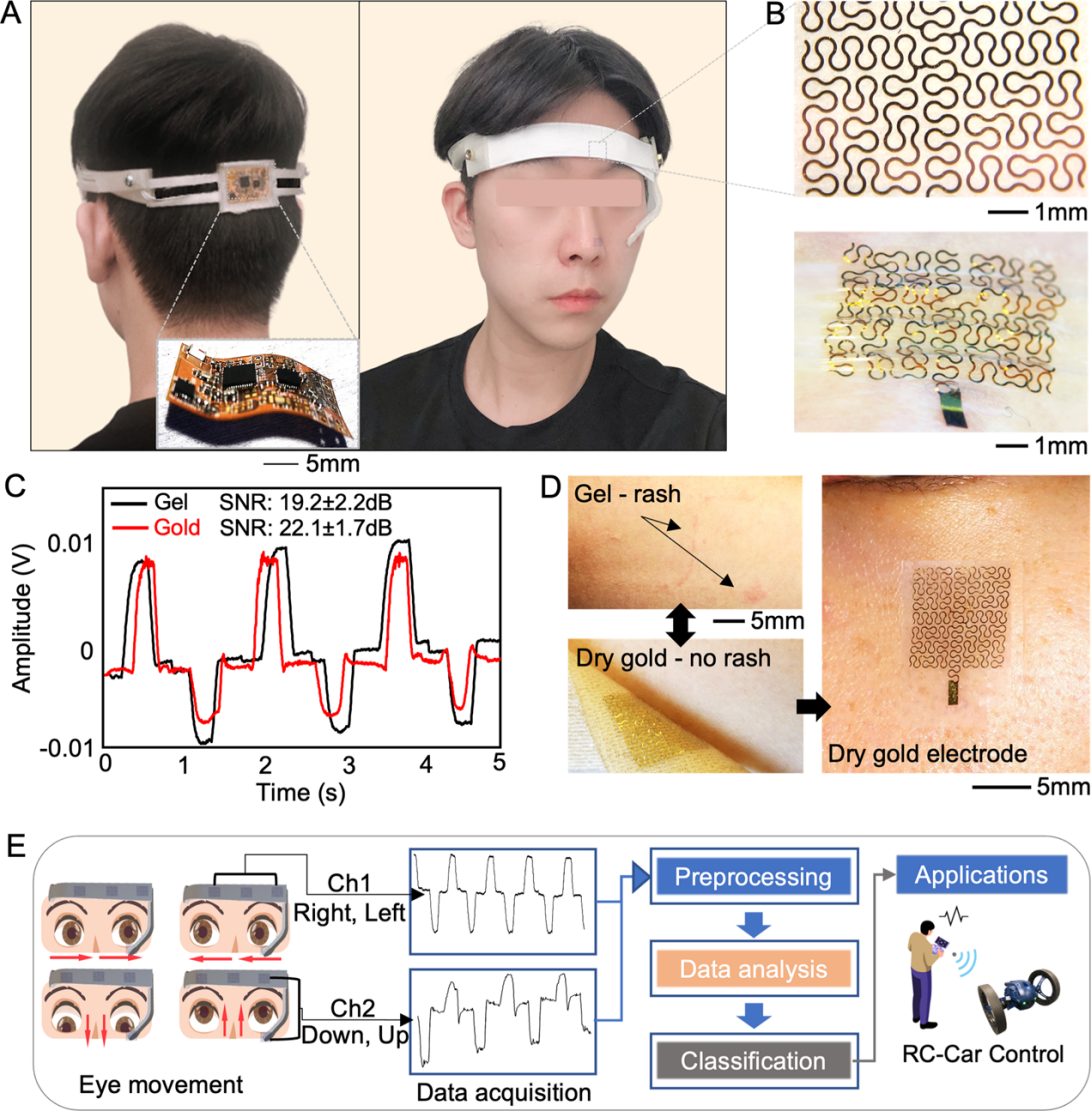
Overview of an integrated bioelectronic system for detecting eye movements and persistent HMI. (A) Photographs of an all-in-one wireless wearable system for EOG-based HMI. (B) Photographs of a skin-like nanomembrane electrode with a zoom-in view (top) and skin-mounted view (bottom) around the eye. (C) Comparison of EOG signals detected by a conventional gel electrode and a dry gold electrode, capturing the higher performance of the dry one. (D) Skin rash after removal of the gel electrode from the skin (top-left) compared to no adverse event for the skin with the dry electrode (bottom-left), continuous mounting of the dry one on the skin for multiple hours (right). (E) Overview of signal processing steps from EOG detection to classification for an example of EOG-based RC car control, as demonstrated in this work.

Figure 1 summarizes the overview of an integrated bioelectronic system for detecting eye movements and persistent HMI. A portable and wearable EOG system enables real-time, continuous, and long-term recording of EOG signals to classify eye movements. Figure 1A shows a subject wearing the headband-type EOG device, integrated with the flexible circuit and fractal gold electrodes. Based on the prior studies,^28−30^ we selected the electrode locations to fit the headband-type platform. Two electrodes were positioned 1 cm above each eye. One electrode was placed 1 cm below the left lower eyelid for vertical eye movement. A common grounding electrode was placed on the middle of the forehead.^29^ The 3D-printed wearable EOG device is composed of a tension string for securing electrodes to the subject’s face. To accommodate various head sizes, thermoplastic polyurethane (TPU), a flexible rubber-like material, makes the headset platform. Figure S1 shows the flexibility of the headband platform. A dry nanomembrane electrode has a stretchable fractal pattern (Figure 1B). The main contribution of this design is to offer maximized stretchability and bending capability without mechanical fracture. The graph in Figure 1C shows EOG signals for left and right eye movements, recorded by two types of electrodes. Then, the calculated signal-to-noise ratio (SNR) compares the performance of the dry gold electrode with the conventional gel electrode.^31^ In the experiment, two electrodes detected changes in EOG amplitudes according to angles of eye direction. The electrodes were positioned 1 cm away from each eye for concurrent comparison. Sensitivity measurements are performed by tracing a series of marked targets, located 60 cm away from the eyes (Figure S2).^14^ The gold electrode’s sensitivity is 12.3 ± 0.5 μV/°, and the conventional gel electrode’s sensitivity is 11.7 ± 0.9 μV/°. The result in Figure 1C shows that the gold electrode (SNR: 22.1 ± 1.7 dB) has a slightly higher SNR than the commercial electrode (SNR: 19.2 ± 2.2 dB), capturing the performance of the dry electrode for high-quality EOG detection. As shown in Figure S3, we compared the skin-electrode contact impedance between a conventional gel electrode and our dry electrode, showing comparable values in the impedance density. In addition, compared to the gel electrode causing skin irritation, the dry gold electrode shows excellent skin compatibility while having intimate contact with the skin (Figure 1D). The overall process that uses EOG signals from eye movements for various applications is well described in Figure 1E. With two electrode channels, we measure EOG data that are preprocessed, filtered, and classified. Figure 1E also shows an example of a radio-controlled (RC) car via EOG, as demonstrated in this work.

### Fabrication and Characterization of a Wearable EOG Device System

**Figure 2:**
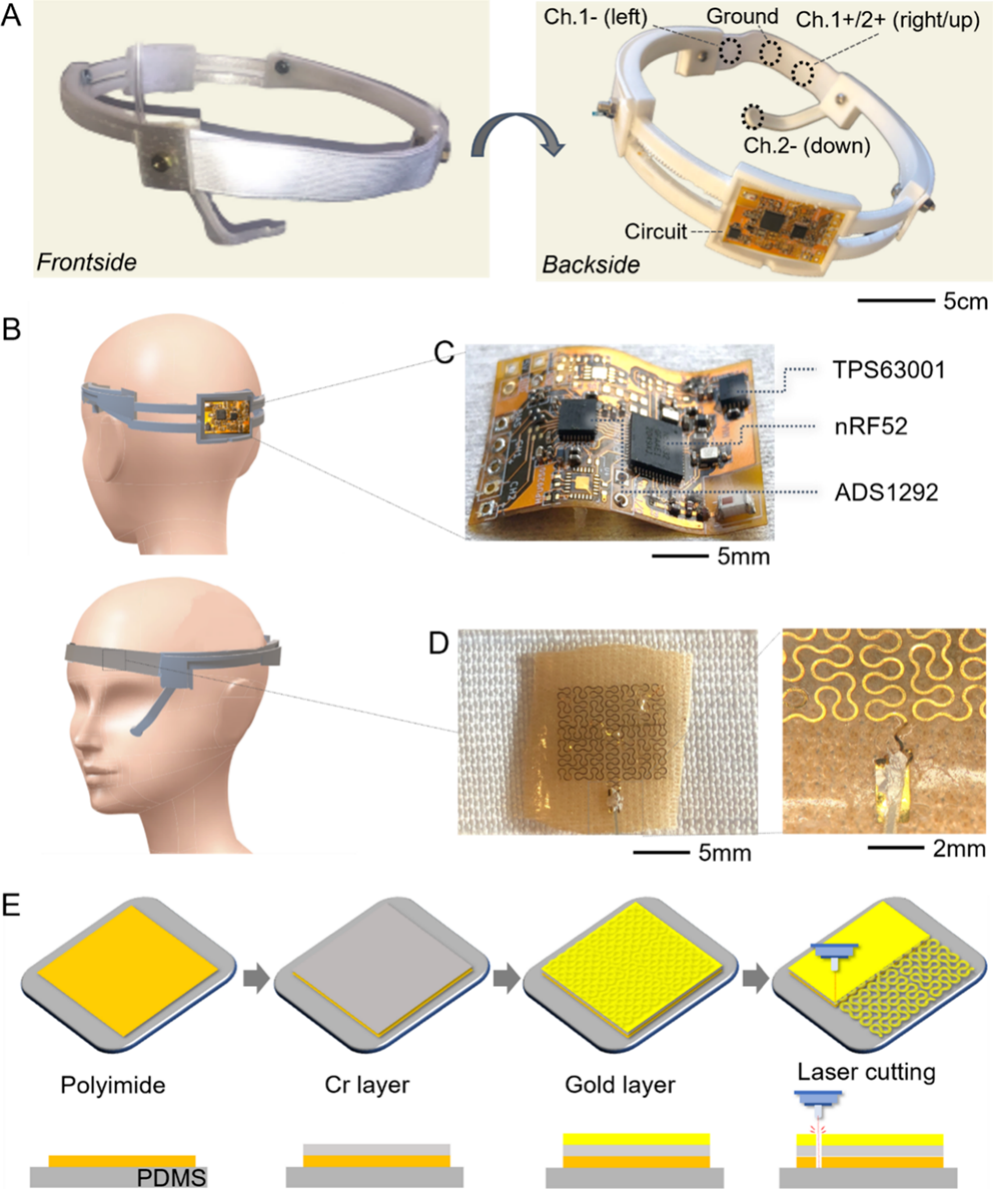
Materials, designs, and fabrication processes of the headband wearable system. (A) Photographs of the fabricated headband EOG system, integrating a flexible wireless circuit and an array of nanomembrane electrodes. (B) Illustrations of a subject who wears the wearable EOG device, showing the exact location of the two-channel electrodes. (C) Photograph of the flexible circuit with integrated chips for EOG signal detection and wireless transmission via Bluetooth. (D) Photographs of the fractal-patterned gold electrode on a soft fabric, connected to the circuit via a thin-film cable. (E) Fabrication processes of the gold electrode using polymer coating, thin-film deposition, and laser cutting.

Recent wearable devices use hard–soft material integration, nanomanufacturing, and chip packaging technologies.^32−34^ In this work, we combine thin-film metallization, laser manufacturing, 3D printing, and system integration to develop a fully integrated all-in-one wearable EOG platform. The base structure uses TPU made by 3D printing, which includes a set of nanomembrane electrodes and a flexible wireless circuit (Figure 2A). A subject can easily wear the headband device with a size-adjustable mechanism (Figure 2B). For wireless signal detection, the system includes a low-profile, flexible circuit having a Bluetooth-low-energy chip and other chip components (Figure 2C; details are given in Figure S4 and Table S2). Time-varying EOG signals are captured by the fractal gold electrodes at 250 Hz and transmitted to the front analog-to-digital converter (ADS1292). Next, to receive sensor data and regulate circuit operation with a built-in microprocessor, the multiprotocol system-on-chip module (nRF52832), which can process and transmit data over 2.4 GHz, is used via the built-in microprocessor. For multiple uses of the wearable device, the flexible circuit contains a rechargeable lithium-polymer battery, charging magnets, and a switch (Figure S5). In addition, the headband system includes a set of fractal gold electrodes that are transfer-printed to the adhesive side of the medical patch (9907T) using a water-soluble tape (Figure 2D).^22,35^ A flexible thin-film cable connects the electrodes with the circuit (Figure 2D). The gold electrodes used in this study were fabricated by following multiple manufacturing steps using a coating of a polymer (polyimide) on a soft PDMS substrate, metallization of Cr and Au, and laser micromachining to create stretchable patterns (Figure 2E).

### Characterization of Mechanical Behavior and Compatibility of the Membrane Electrodes

**Figure 3:**
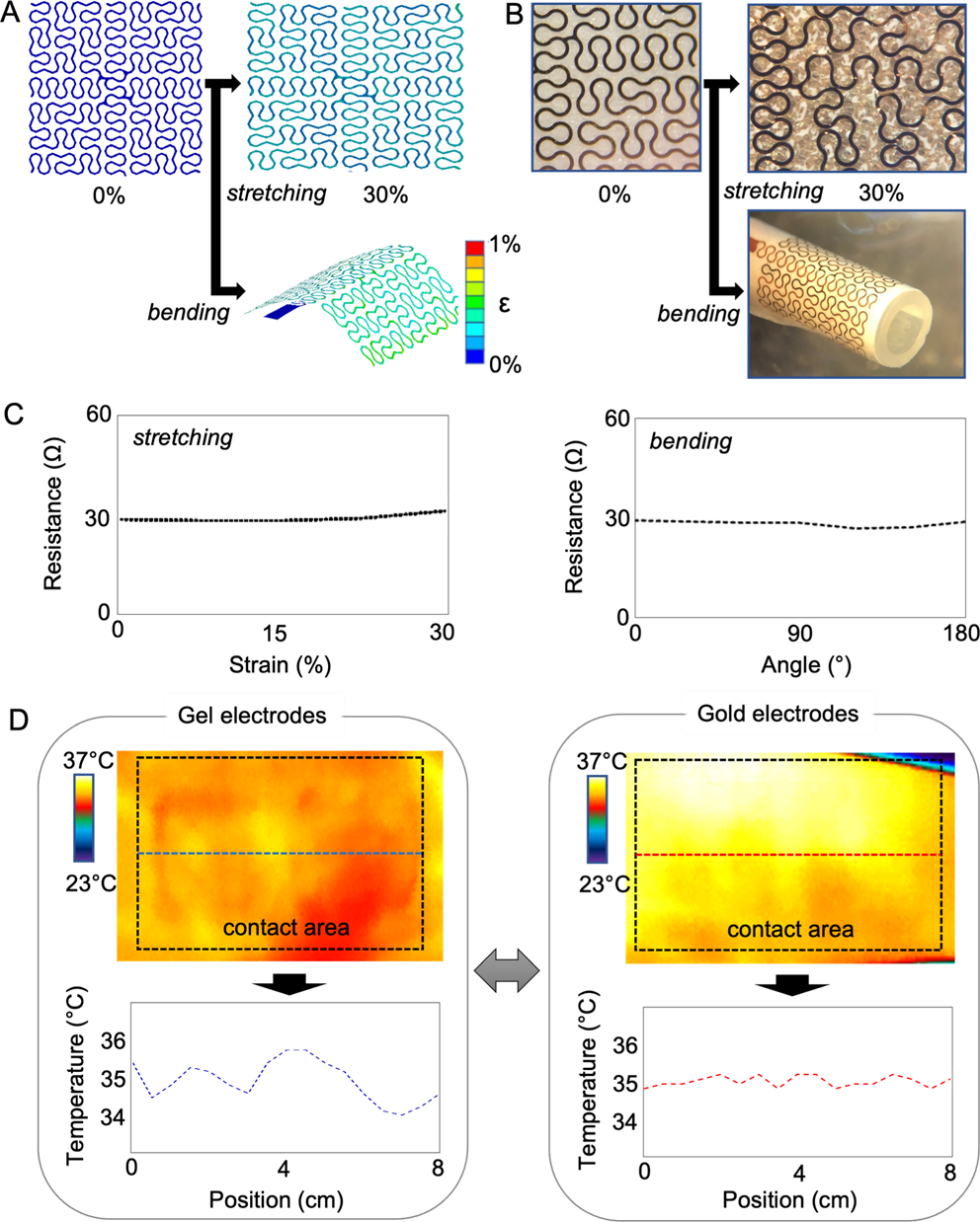
Characterization of mechanical behavior and compatibility of the membrane electrodes. (A) Computational study results of mechanical behavior estimation of an electrode with stretching and bending. (B) Experimental validation of the electrode’s reliability with stretching and bending. (C) Resistance measurements of the electrode to quantify the reliability, showing negligible changes during stretching and bending. (D) Comparison of skin biocompatibility of a gel electrode (left) and a gold dry electrode (right), showing that the dry electrode has no side effects while the gel electrode causes skin irritation and elevates temperature.

The mechanical reliability of stretchable electrodes is critical to maintaining the skin-contact quality during real-time continuous EOG detection. Therefore, we conducted a set of computational studies using finite element analysis (FEA), considering cyclic stretching and bending situations when an electrode is mounted on the skin. Figure 3A shows the FEA results of an electrode, showing that the maximum principal strain applied to Au is less than 1% under the tensile and bending strain. The fractal-patterned design was used to manufacture electrodes, and we validated the mechanical reliability (Figure 3B). A microscopic investigation observes mechanical fractures before and after stretching and bending tests, showing no visual damage. In this test, the maximum tensile strain was applied up to 30%, and the bending angle was 180° with a radius of curvature: 6 mm. We chose 30% strain based on prior studies showing that a human’s exterior epithelial tissue can be stretched up to 20% without damage,^36^ and normal skin deformations do not exceed the selected bending curvature.^37^ The visual observation of mechanical fracture was further validated by measuring electrical resistance. Figure 3C shows the negligible resistance changes during the electrode stretching and bending. Furthermore, we investigated the skin biocompatibility of a gel electrode and a dry gold electrode using infrared thermography (Figure 3D). While the dry electrode shows no side effects after 8 h of wearing, the gel electrode causes skin irritation and temperature elevation after 4 h (Figure S6). When using the gel electrode, adhesive pads mounting the rigid electrode to the skin remove dead skin cells from the epidermis, causing skin rashes.

### Optimization of Real-Time Classification via Signal Processing and Feature Extraction

**Figure 4:**
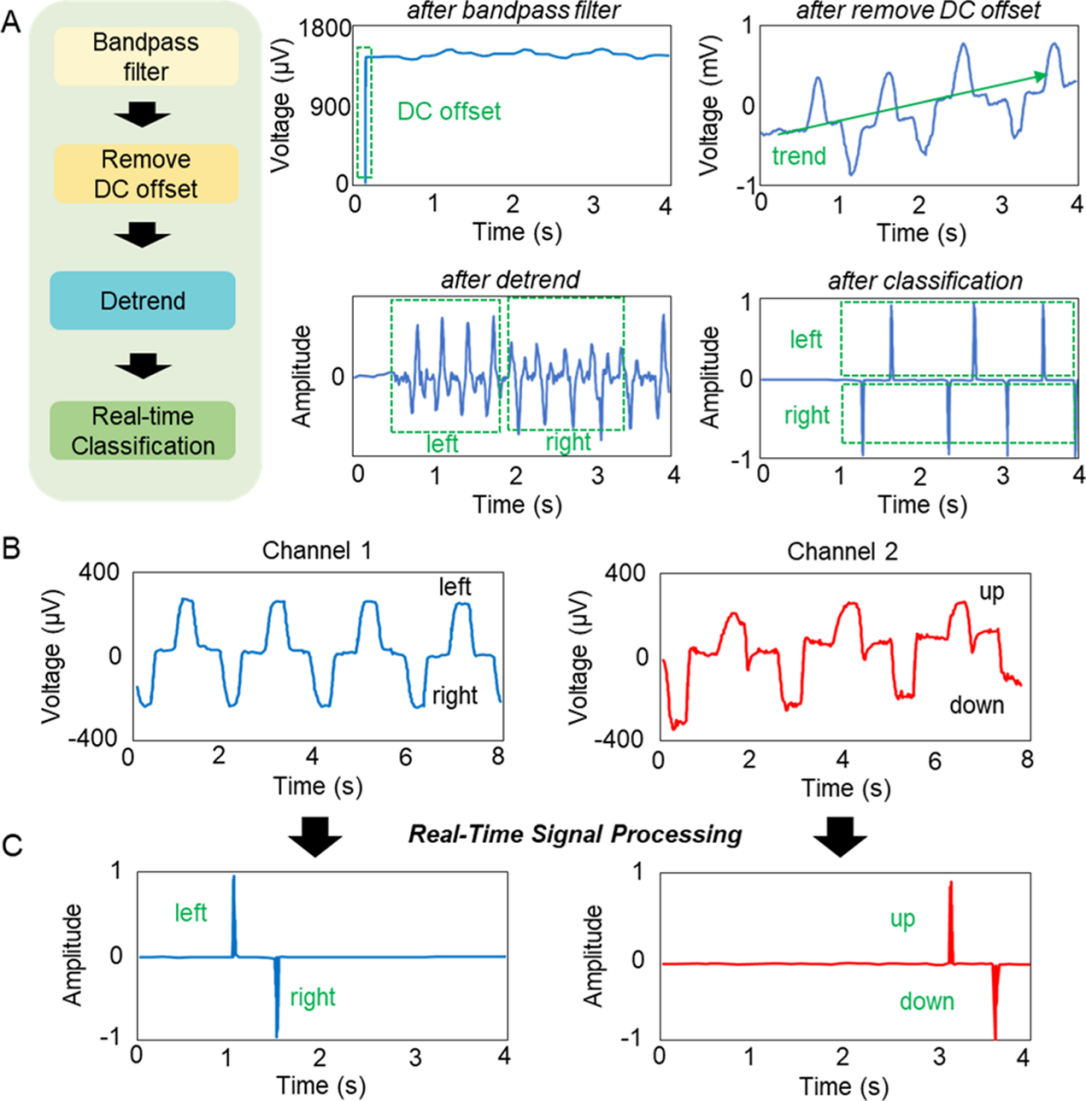
Optimization of signal processing and feature extraction for real-time data classification. (A) Step-by-step sequence of processing of measured EOG signals from the wearable device; four different graphs on the right show examples of processed signals after each step, including bandpass filter, remove DC offset, detrend, and classification. (B) Representative examples of raw EOG signals and corresponding eye movements, including left, right, up, and down motions. (C) Set of classified signals after filtering and feature extraction.

A flow chart in Figure 4A shows a step-by-step sequence of processing of measured EOG signals from the wearable device; four corresponding graphs on the right show examples of processed signals after each step, including bandpass filter, DC offset, detrend, and classification. In this process, the EOG raw data are received by a Python program through Bluetooth. Since EOG data mainly contain low frequencies (sampling rate: 250 Hz), a third-order Butterworth finite impulse response filter (FIR) is used to remove noise.^38^ FIR is a bandpass filter widely used in digital signal processing, showing an excellent linear phase character.^39^ To remove the DC offset, the first offset value is removed from others. As a result, measured signals can show trends that are not intrinsic to the data. To eliminate this trend, detrend function is used. Lastly, filtered data from three different steps are classified by evaluating the magnitude. The classified data are converted into a signal with a size of 1, and the direction of the eye is classified according to the code. A set of representative EOG signals in Figure 4B show raw data from four different eye movements. Among them, the horizontal direction of the eyes is channel 1, and the vertical direction of the eyes is channel 2. After signal processing, these signals are classified as left, right, up, and down motions (Figure 4C). A real-time demonstration in Video S1 shows how this process works.

### Development and Comparison of Machine Learning Algorithms for Data Classification

**Figure 5:**
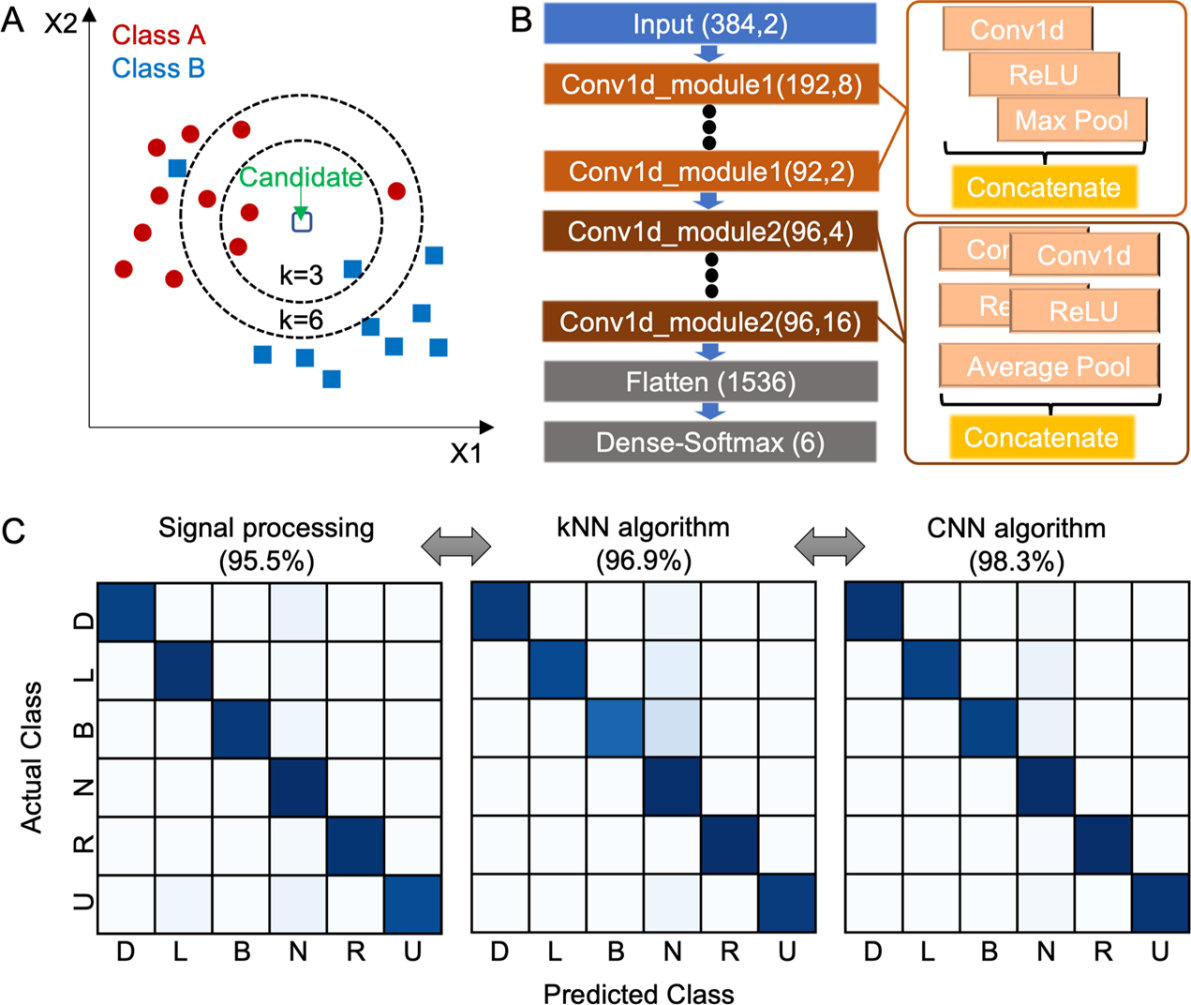
Development and comparison of machine learning algorithms for data classification. (A) Illustration of an example of a kNN classifier. (B) Flowchart showing a spatial CNN model with filters of decreasing size, flattening, and followed by the dense–softmax output. (C) Comparison of data classification results, showing three different confusion matrices, including the signal processing outcome (left), kNN algorithm (middle), and CNN algorithm (right); the highest accuracy is achieved by the CNN method. In each confusion matrix, D means down, L means left, N means null, R means right, U means up, and B means blink.

Prior studies show the limitation of signal processing when detecting more than five classes;^28^ with six classes, the accuracy was only 91.25%. According to other studies, a kNN algorithm is more efficient when classifying EOG signals than decision tree and support vector machine methods.^40,41^ The kNN classification uses the nearest distance metric and the neighbor’s number *k* value. When one of the parameters is varying, another parameter is fixed.^41,42^ In this kNN algorithm, testing data are classified by finding the greatest number, with the closest relative distance to neighbors; each neighbor belongs to a specific class. Figure 5A shows an example of a kNN classification where the test candidate is classified as either blue squares or red circles. If *k* = 3, the candidate is assigned to the red circles (2 red circles > 1 blue square). If *k* = 6, the candidate is again assigned to the red circles (5 red circles > 1 blue square). To compare the performance of machine learning algorithms, we also developed a CNN classifier. The details of the overall CNN classification processes are shown in Figure 5B. The CNN model featuring layers of one-dimensional convolutions consists of two kinds of modules. Then, this model is followed by filters of flattening and a dense–softmax output. In this study, EOG data collected by the wearable device are split into the training set (75%) and the test set (25%). The preprocessed data are transferred to either the kNN or the CNN classifier. Then, the test data set is analyzed by comparing the training data set. Each model predicts the test results and shows the results through the confusion matrixes. Figure 5C summarizes and compares the performance of signal processing and two machine learning methods. There are multiple eye movements, including up (U), down (D), left (L), right (R), blink (B), and null (N), used in this study. The signal processing method with six classes shows an accuracy of 95.5%. Compared to that, kNN and CNN methods with six classes show higher accuracies, 96.9 and 98.3%, respectively. Confusion matrixes from signal processing, kNN, and CNN algorithms including accuracies of each class are shown in Figure S7. Overall, the CNN classification result shows the highest accuracy among reported articles that detect EOG signals. Also, it takes less than a second from pre-processing to real-time classification. Table 1 captures the advantages of our wearable system and superior classification performance compared to prior studies. Table 1 also shows that our wearable system is compact and flexible by comparing previous EOG devices based on the size and type of circuits.

**Table 1:** Comparison of Wearable EOG Devices for Classification of Eye Movements and HMI Applications

references	circuit type	form factor (cm × cm)	integrated single device	electrode type	no. of electrodes	no. of classes	classification accuracy (%)	platform	HMI application
this work	flexible	2 × 3	yes	dry, membrane (gold)	4	6	98.3	headband with a flexible circuit	two-wheeled RC car
(14)	rigid, bulky	12 × 15	no	dry (gold)	5	4	94	wire-connection with a circuit	wheel-chair
(12)	rigid, bulky	6 × 6	no	dry (gold)	5	5	92	wire-connection with a circuit	drone helicopter
(43)	rigid, bulky	4 × 6	no	dry, (graphene)	3	5	98	headband with rigid circuit	RC car
(23)	rigid, bulky	5.5 × 8	no	dry, (graphene)	3	2		headband with rigid circuit	LED array
(44)	rigid, bulky	6 × 6	no	dry (Ag/AgCl)	5	4		eyeglass	
(45)	rigid, bulky	5 × 5	no	dry (Ag/AgCl)	4	1		headband with rigid circuit	
(46)	rigid, bulky	3 × 20	no	dry (silver)	3	2		headband with rigid circuit	
(47)	rigid, bulky	5 × 7	no	gel (Ag/AgCl)	5	5	75.5	eyeglass	omnidirectional-robot
(48)	rigid, bulky	4 × 8	no	gel (Ag/AgCl)	5	5	97	armband	game
(49)	rigid, bulky	11 × 16	no	gel (Ag/AgCl)	5	5	89	helmet	keyboard
(28)	rigid, bulky	4 × 8	no	gel (Ag/AgCl)	4	6	91.3	rigid headband	wheelchair + keyboard

### Demonstration of Wireless Real-Time Control of a RC Car with the Wearable Device

**Figure 6:**
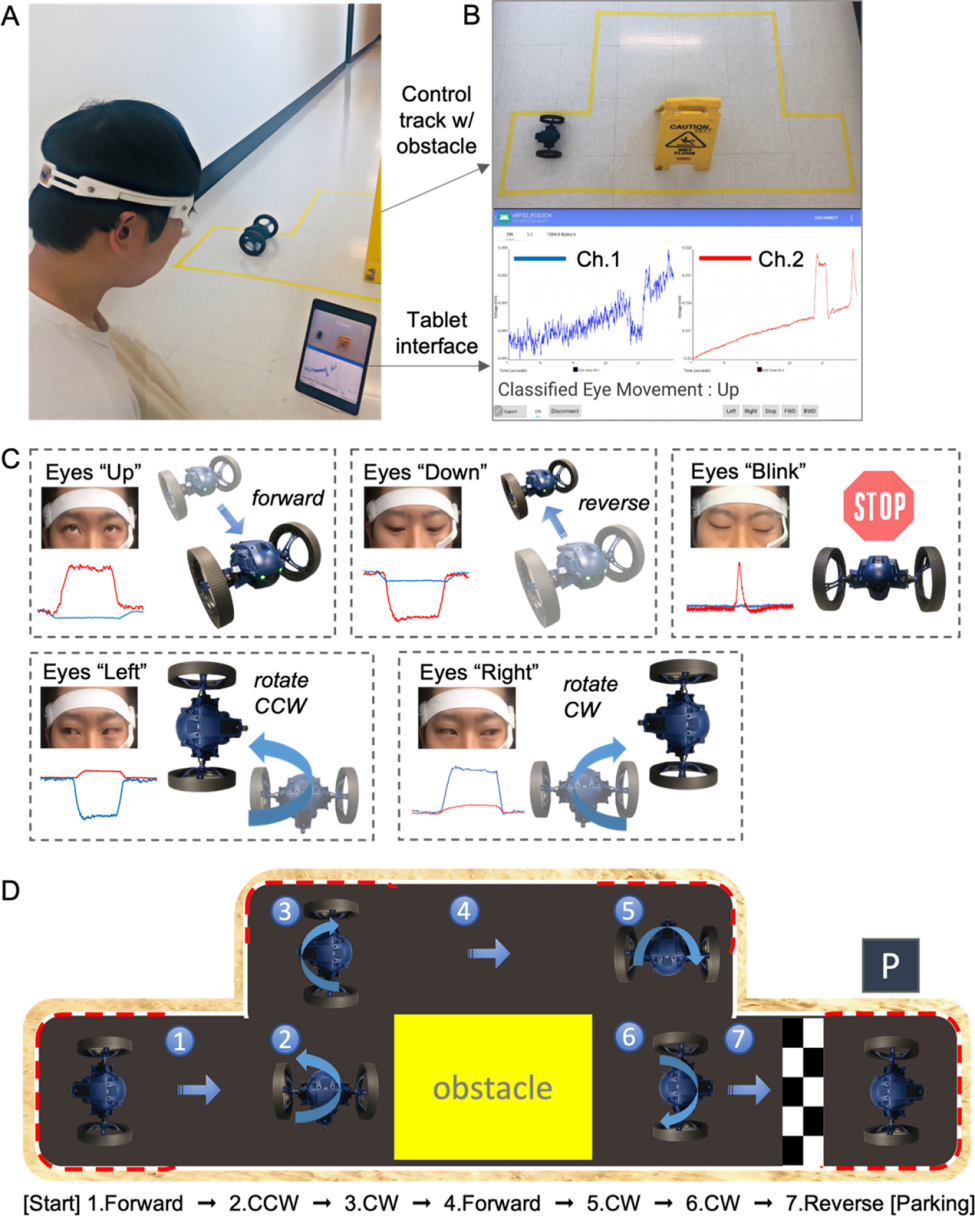
Demonstration of wireless real-time control of a two-wheeled RC car with the wearable device. (a) Photograph showing a subject who wears the wearable EOG device, a tablet capturing the real-time EOG signals, and a two-wheeled RC car to control. (b) Top photograph showing a control track with an obstacle that the car follows, while the bottom photograph showing the zoomed-in view of an Android app for displaying EOG signals and real-time classification outcomes. (c) Five different control commands used for the car, including up, down, blink, left (CCW; counter-clockwise), and right (CW; clockwise) motions. (d) Top-view photograph capturing the pathway that the car follows while avoiding the obstacle; in this demonstration, seven commands are used from starting to parking.

In this work, we demonstrate an example of persistent wireless HMI using the headband wearable device and EOG signals (Figure 6). Multiple eye movements, detected by sensors, could successfully control a two-wheeled RC car by accurately following the designated pathway and avoiding an obstacle. Figure 6A captures a photograph showing a subject who wears the sensor-integrated headband, a tablet capturing the real-time EOG signals, and a two-wheeled RC car to control. In Figure 6B, the top photograph shows a control track with an obstacle that the car follows, while the bottom photograph captures the zoomed-in view of an Android app for displaying EOG signals and real-time classification outcomes. In this demonstration, we use five different control commands, including up, down, blink, left (CCW; counter-clockwise), and right (CW; clockwise) motions (Figure 6C). Considering an emergency case during operation, the blink command immediately stops the car when unintended eye movements are classified. The two-wheeled RC car follows eye movements of a subject who wears the device, which moves the car from the starting position to the parking location. Seven consecutive commands are delivered to the car (Figure 6D), including (1) go forward, (2) CCW rotation and go forward, (3) CW rotation, (4) go forward, (5) CW rotation and go forward, (6) CW rotation, and (7) go reverse to park. The real-time control of this car using eye movements appears in Video S2.

## Conclusions

This paper reports a comprehensive set of studies that develop a soft headband bioelectronic system and persistent HMI using EOG signals. The wearable headband platform offers a firm contact of stretchable electrodes with the skin, which also can be worn by different users with various head sizes. A simplified manufacturing process, including metal deposition and laser cutting, fabricates an array of thin-film dry electrodes without needing conductive gels for high-quality EOG recording. The highly stretchable and flexible electrode shows reliability in cyclic mechanical tests while demonstrating excellent skin compatibility over 8 h. The fractal-patterned gold electrode could be repeatedly used throughout this study, but quantification of the reusability of the electrodes will be included in future work. Measured EOG signals are filtered and classified by a signal processing method and kNN and CNN algorithms. Among them, the CNN-based classification shows the highest accuracy of 98.3% with six classes. Demonstration of wireless real-time control of a two-wheeled RC car captures the performance of the wearable device for persistent HMI. In this study, seven commands using eye movements could successfully control a car on a confined track while avoiding an obstacle. Future studies will address limitations, such as crosstalk between vertical and horizontal channels or EEG and EMG signals.

## Experimental Section

### Fabrication of the Integrated Wearable System

The wearable EOG device consists of a fractal gold electrode, headband-type platform, and flexible circuit. PDMS (Sylgard 184, Dow) was spin-coated on a clean glass slide. An 8.47 μm thick polyimide sheet (Kapton film, DuPont) was laminated onto the PDMS-coated glass slide first, followed by a 5 nm thick Cr layer and 200 nm thick Au layer that was deposited using an electron beam deposition tool (Denton Explorer), respectively. We studied open-mesh structured fractal patterns (a bending radius of 0.39 mm and a trace width of 0.16 mm). The fractal pattern was cut by a femtosecond IR laser micromachining tool (WS-Flex, Optec), which is a multi-purpose, high-precision processing tool for various materials. The cut fractal pattern was transferred using the water-soluble tape (ASWT-2, Aquasol) from the PDMS. The wearable 3D headband platform was designed by SolidWorks and printed by a 3D printer (Cubicon Single Plus 3DP-310F) with TPU filaments (Cubicon TPU Filament). TPU filaments are flexible with superior strength. We designed the headband-type platform that can be resized according to the head size through the tension string and the auxiliary equipment (Figure S8). Chip components on the flexible circuit were soldered to the plate with a solder paste (SMDLTLFP10T5, Chip Quik) and then heated at 100 °C. The set temperature increased by 10 °C every minute to a final temperature of 150 °C. A small lithium polymer battery (capacity: 40 mA h, Digi-Key) was modified to allow for easy charging by connecting two charging magnets and a switch to the battery. The circuit with a 40 mA h battery lasted 5.1 h, which is around 8 mA power consumption.^31^ The flexible circuit was attached to the back of the headband-type platform. The fractal gold electrodes were connected to the circuit via encapsulated ACF wires. Lastly, the electrodes were attached to the tension string.

### Finite Element Analysis

This work includes the results of studied FEA to investigate a fractal gold electrode’s mechanical behaviors using commercial software (ANSYS). This analysis focused on the mechanical fracture of the electrode upon cyclic bending and stretching. The modeling analyzed the maximum principal strain in the electrode consisting of three layers: an 8.47 μm thick polyimide sheet, 5 nm thick Cr layers, and 200 nm thick Au layers (Figure S9). Table S1 shows the details of the material properties (Young’s modulus and Poisson’s ratio). One side of the substrate is fixed as a support fix, and the other side is moved using the displacement function. The boundary conditions were applied to the Ecoflex substrate.

### Experimental Study of Mechanical Behavior

A customized stretcher conducted the axial stretching test. Two clamps held the sample. The strains were determined by controlling the distance from 0 to 30%. The bending test was conducted manually by a rigid circular cylinder. The bendability from 0 to 180° of the fractal gold electrodes was assessed manually with a bending radius of 6 mm (details are given in Figure S10). A digital multimeter is used to measure and record a resistance change on the fractal gold electrode.

### Data Acquisition and Training

To detect EOG signals, two electrodes were positioned 1 cm above each eye. One electrode was placed 1 cm below the left lower eyelid for vertical eye movement. A common grounding electrode was placed on the middle of the forehead (Figure S11). Before obtaining the data, the skin (electrode position) was optionally wiped with alcoholic cotton to remove foreign matter. The eyes were moved in six eye movements (left, right, up, down, blink, and null), and the gaze was within 1 s. The raw EOG signals from the wearable EOG device were measured and recorded by an Android tablet via Bluetooth. The custom Android application simultaneously transmits and exports data for channels 1 and 2. As shown in Figure S12, MATLAB labeled the acquired EOG data to train the CNN classifier. Then, the EOG data were trained and modeled by a machine learning algorithm and TensorFlow platform. The modeled file classifies the subject’s EOG signals in real-time through the machine learning interface and TensorFlow platform in the Android application.

### Analysis of the SNR

The experiment was conducted by looking left and right three times at regular intervals for 5 s in this recording. The raw data were recorded into five s segments (five total). This analysis involves the measurement of EOG signal size and removal of the average value of the EOG signal using the following equation: SNR (dB) = 10 Log_10_(rms_signal/rms_noise). The results and the standard error were calculated as an average over the number of recordings.

### Human Subject Study

The human pilot study involved multiple healthy volunteers; the study followed the approved IRB protocol from the Georgia Institute of Technology (no. H20226). All participants agreed and signed the consent form to allow the experiment procedure.

## Supporting Information Available

Device bending test; experimental setup for EOG sensitivity; impedance measurement with electrodes; functional chips used in the flexible wireless circuit; battery components; skin rash test; comparison of the detailed confusion matrix; wearable EOG device assembly; FEA simulation modeling; tensile and bending tests; locations of the dry gold electrodes; and raw EOG data and classified result (PDF)
Monitoring of EOG (MP4)
Demonstration of a mini-drone car (MP4)
