# Supplementary material for: Soft Wireless Headband Bioelectronics and Electrooculography for Persistent Human–Machine Interfaces
Source: ACS Appl Electron Mater. 2023 Feb 8;5(2):877–86. doi: 10.1021/acsaelm.2c01436 (PMC9979786; doi:10.1021/acsaelm.2c01436)

Supporting Information

Soft Wireless Headband Bioelectronics and Electrooculography for Persistent Human-Machine Interfaces

Seunghyeb Ban[1,2,10], Yoon Jae Lee [2,3,10], Shinjae Kwon[2,4], Yun Soung Kim[5], Jae Won Chang[6,], Jong-Hoon Kim[1,7*], Woon-Hong Yeo[2,4,8,9*]*

[1]School of Engineering and Computer Science, Washington State University, Vancouver, WA, 98686, USA

[2]IEN Center for Human-Centric Interfaces and Engineering at the Institute for Electronics and Nanotechnology, Georgia Institute of Technology, Atlanta, GA 30332, USA

[3]School of Electrical and Computer Engineering, Georgia Institute of Technology, Atlanta, GA 30332, USA

[4]George W. Woodruff School of Mechanical Engineering, College of Engineering, Georgia Institute of Technology, Atlanta, GA 30332, USA

[5]BioMedical Engineering and Imaging Institute, Icahn School of Medicine at Mount Sinai, New York, NY 10029, USA

[6]Department of Otolaryngology Head and Neck Surgery, School of Medicine, Chungnam National University Hospital, Daejeon 35015, Republic of Korea

[7]Department of Mechanical Engineering, University of Washington, Seattle, WA, 98195, USA

[8]Wallace H. Coulter Department of Biomedical Engineering, Georgia Institute of Technology and Emory University School of Medicine, Atlanta, GA 30332, USA

[9]Parker H. Petit Institute for Bioengineering and Biosciences, Institute for Materials, Neural Engineering Center, Institute for Robotics and Intelligent Machines, Georgia Institute of Technology, Atlanta, GA 30332, USA

[10]These authors contributed equally to this work.

*E-mail: Woon-Hong Yeo: whyeo@gatech.edu, Jong-Hoon Kim: jh.kim@wsu.edu or Jae Won Chang: strive1005@cnuh.co.kr

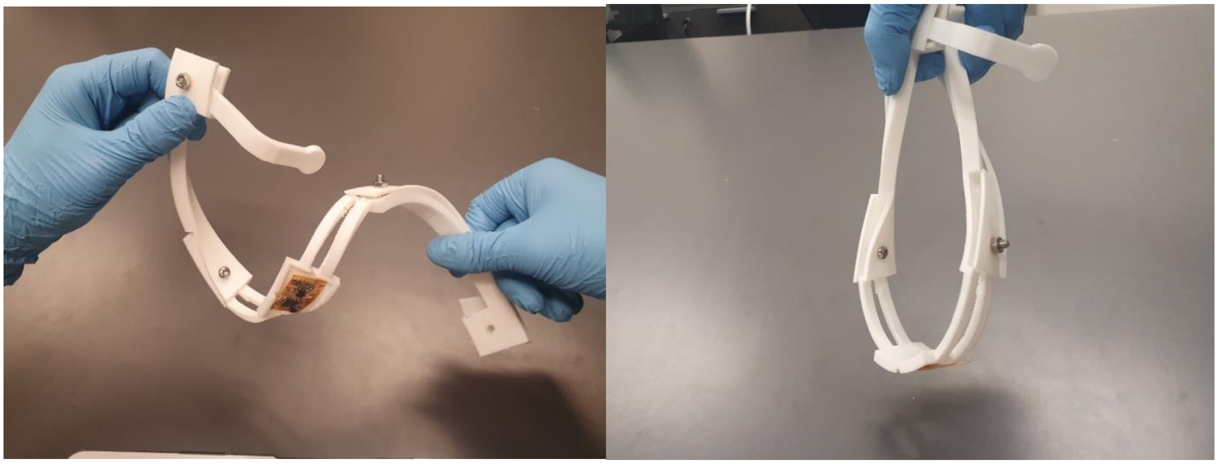

Figure S1. Photo (left) of twisted headband platform and photo (right) of banded headband platform.

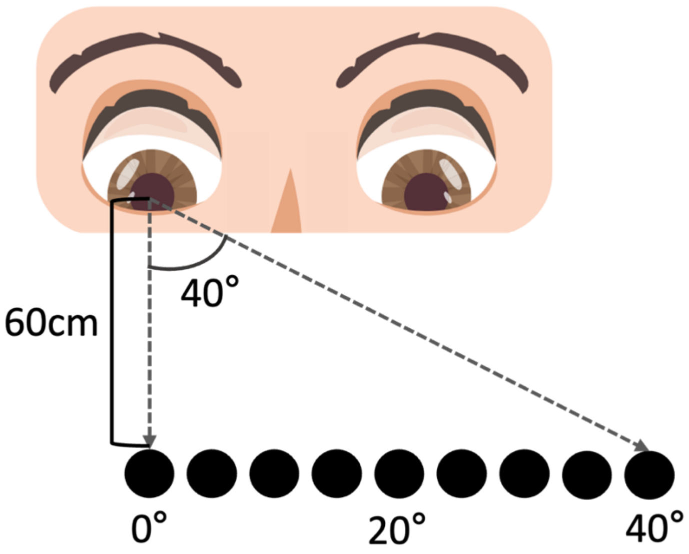

Figure S2. An experimental setup for EOG sensitivity by tracing a series of marked targets.

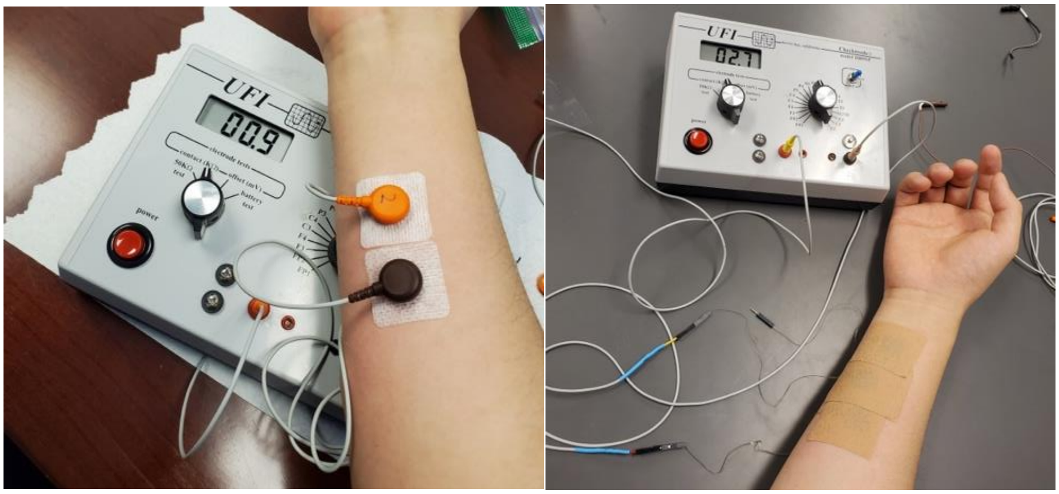

	Impedance (Ohm)	Unit area (mm^2)	Impedance density (Ohm / cm^2).
Gel electrode	0.9	113	0.80
Dry gold electrode	2.7	35	0.95

Figure S3. Photos of impedance measurement with conventional gel electrodes (left) and of dry gold electrodes (right).

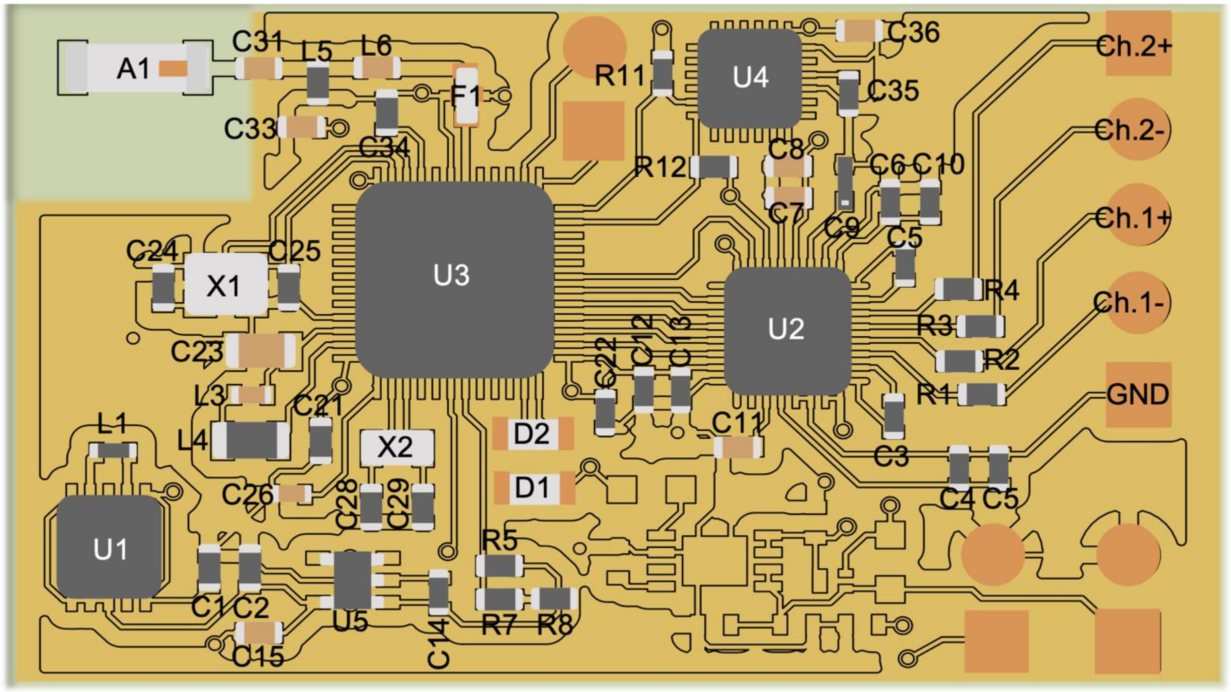

Figure S4. List of functional chips used in the flexible wireless circuit.

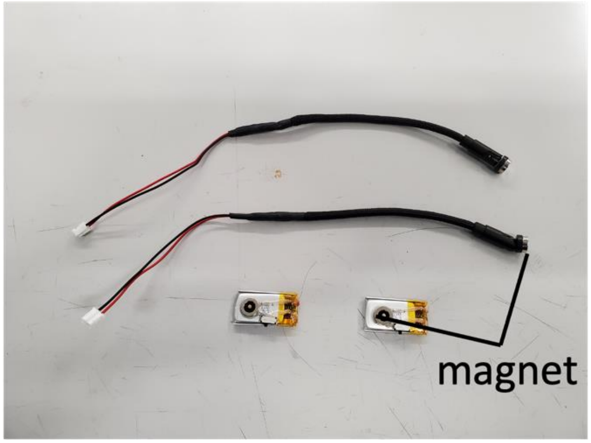

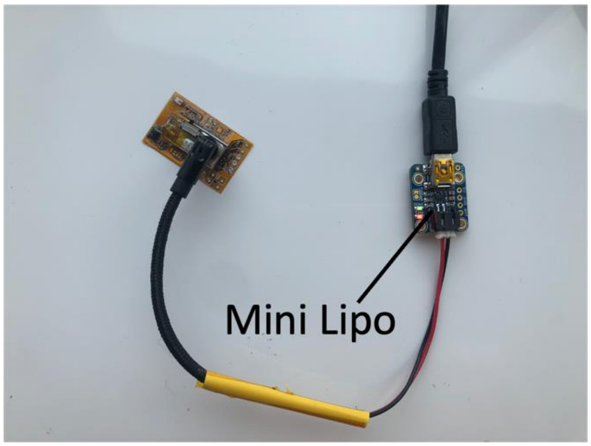

Figure S5. Photo of batteries and charger cables (left) and a charging circuit (right).

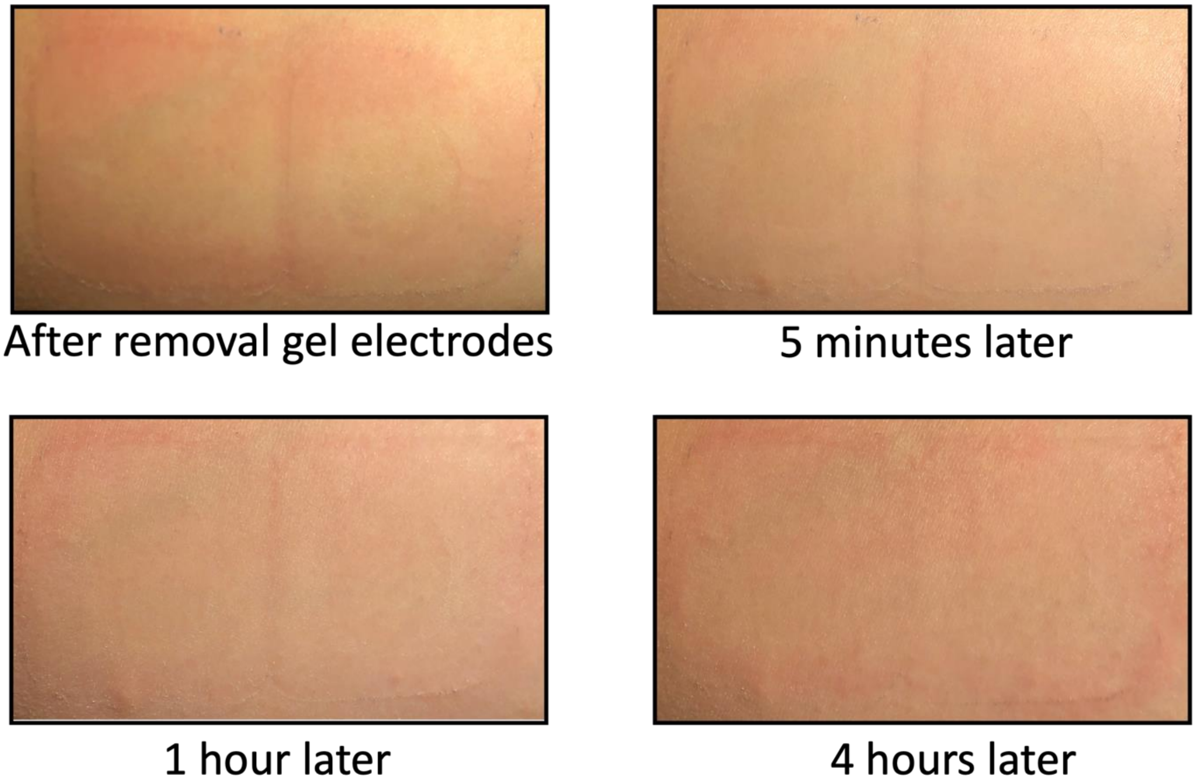

Figure S6. Photographs of skin rash to show How long a rash last.

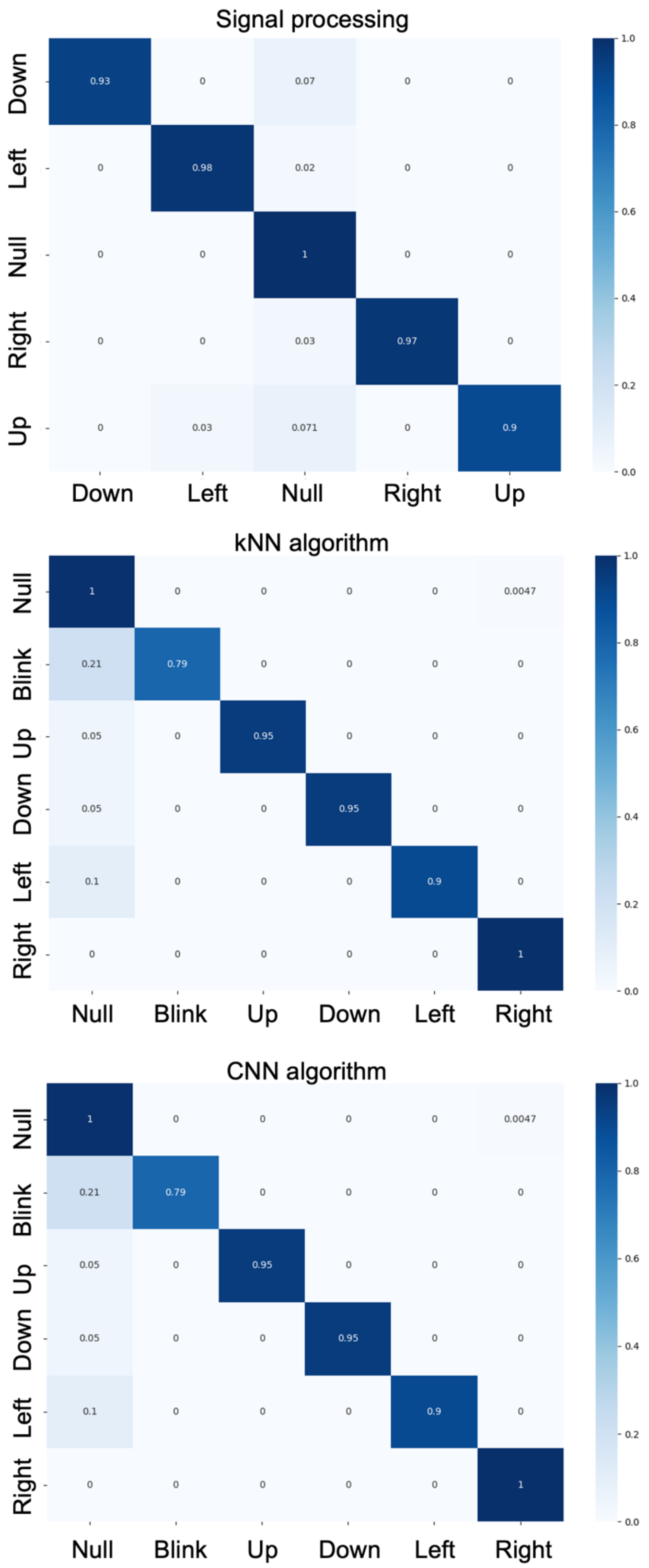

Figure S7. Comparison of detailed confusion matrix from signal processing, kNN, and CNN algorithms.

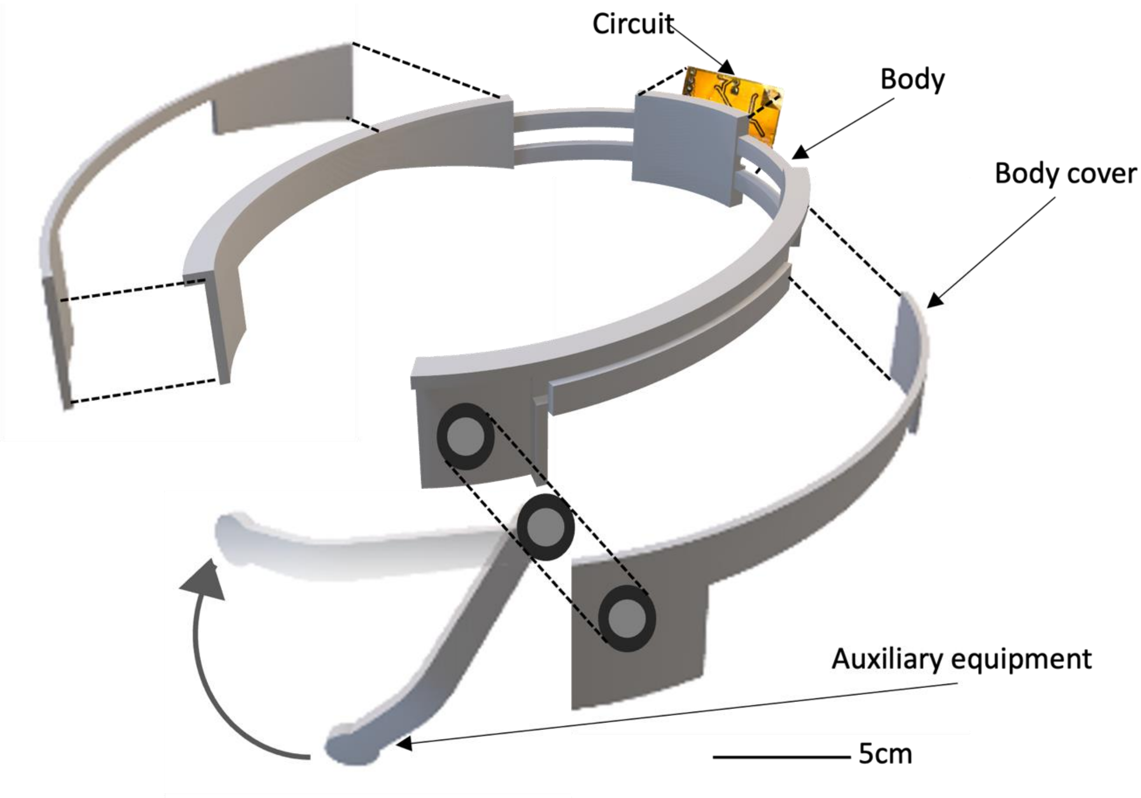

Figure S8. Exploded view of assembly drawing.

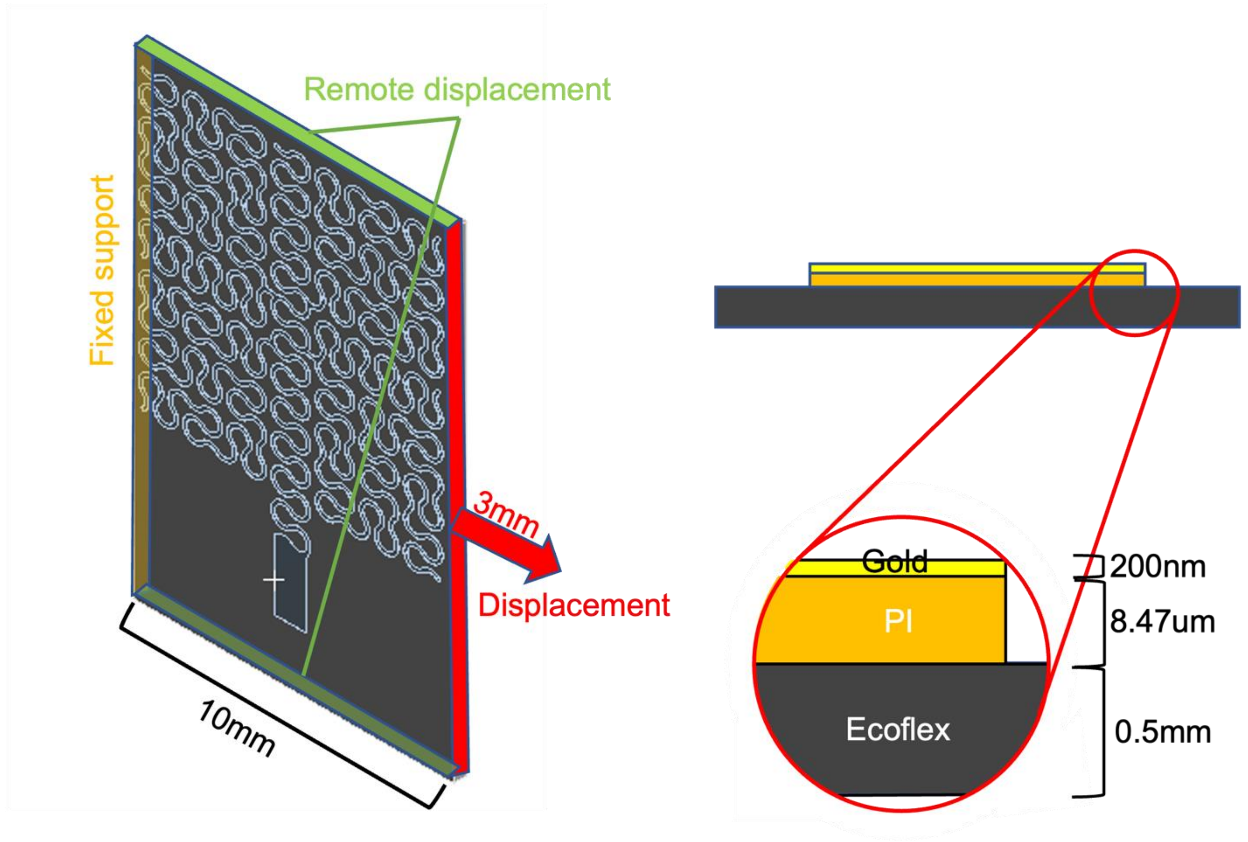

Figure S9. Setting FEA simulation value in ANSYS.

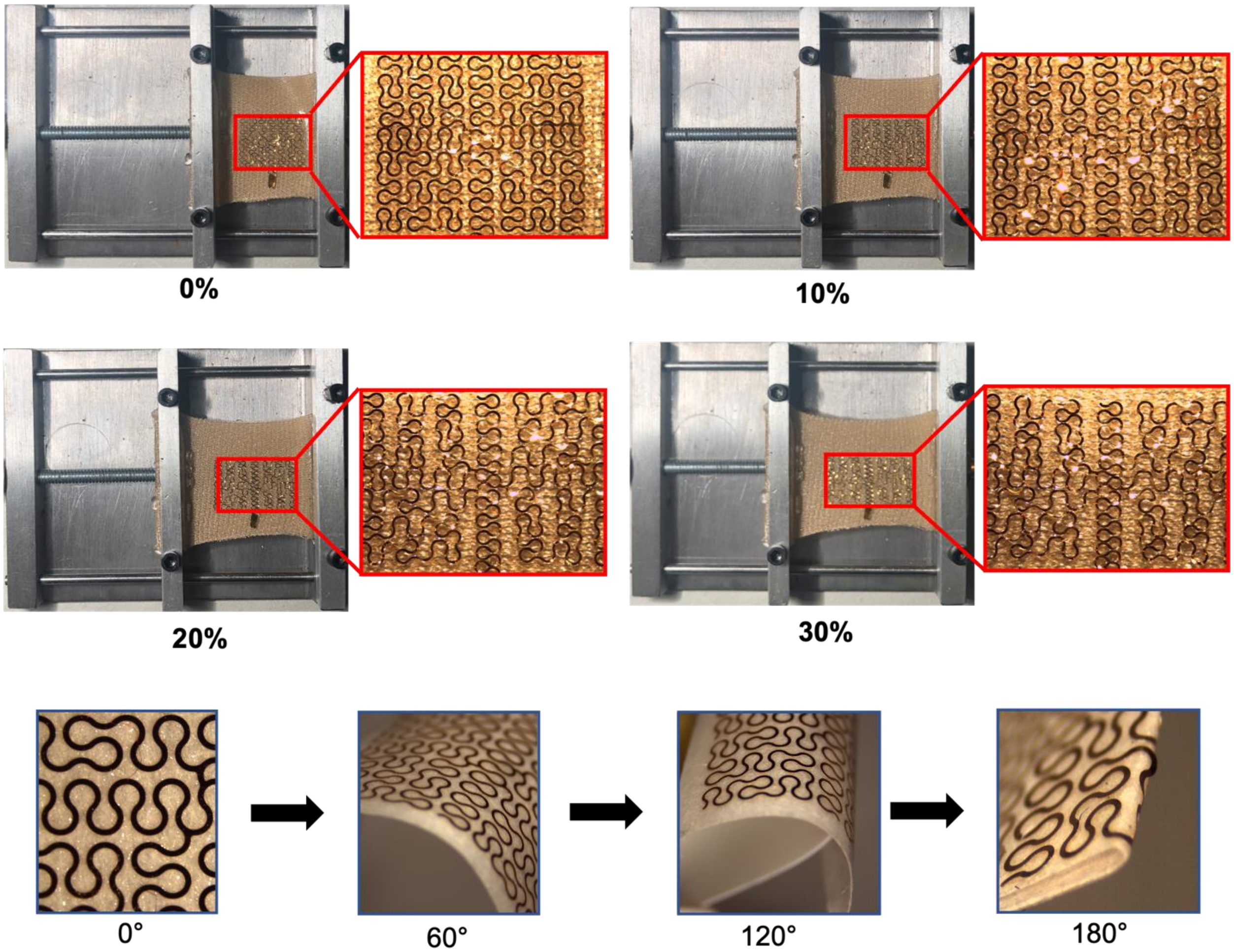

Figure S10. Photos (Up) of tensile testing of the exploded view of the gold fractal electrode. Photos (Down) of bending testing of the exploded view of the gold fractal electrode.

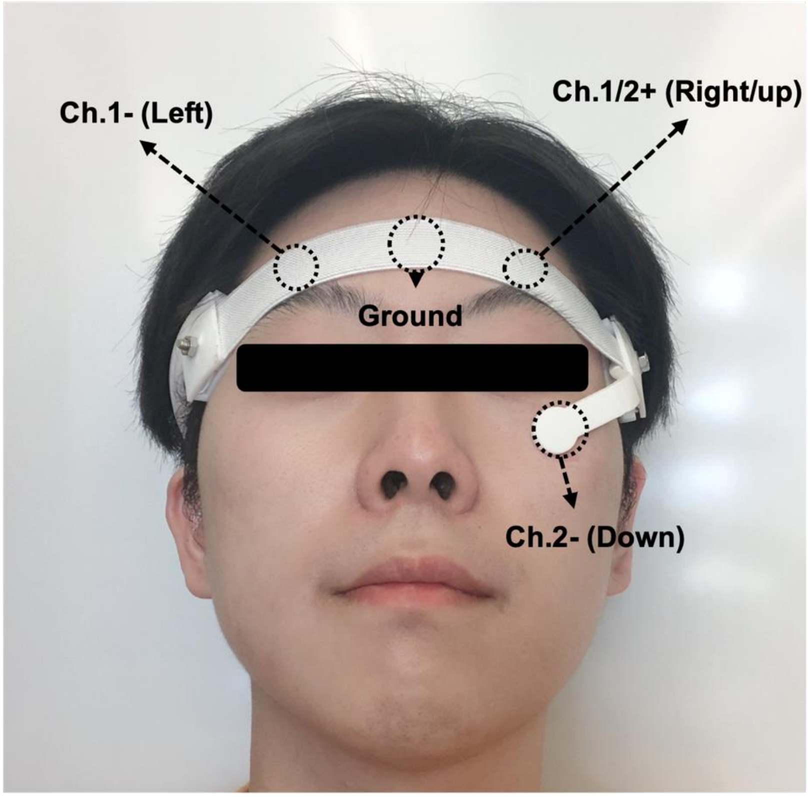

Figure S11. Photo of locations of the dry gold electrodes placed on a subject's facial.

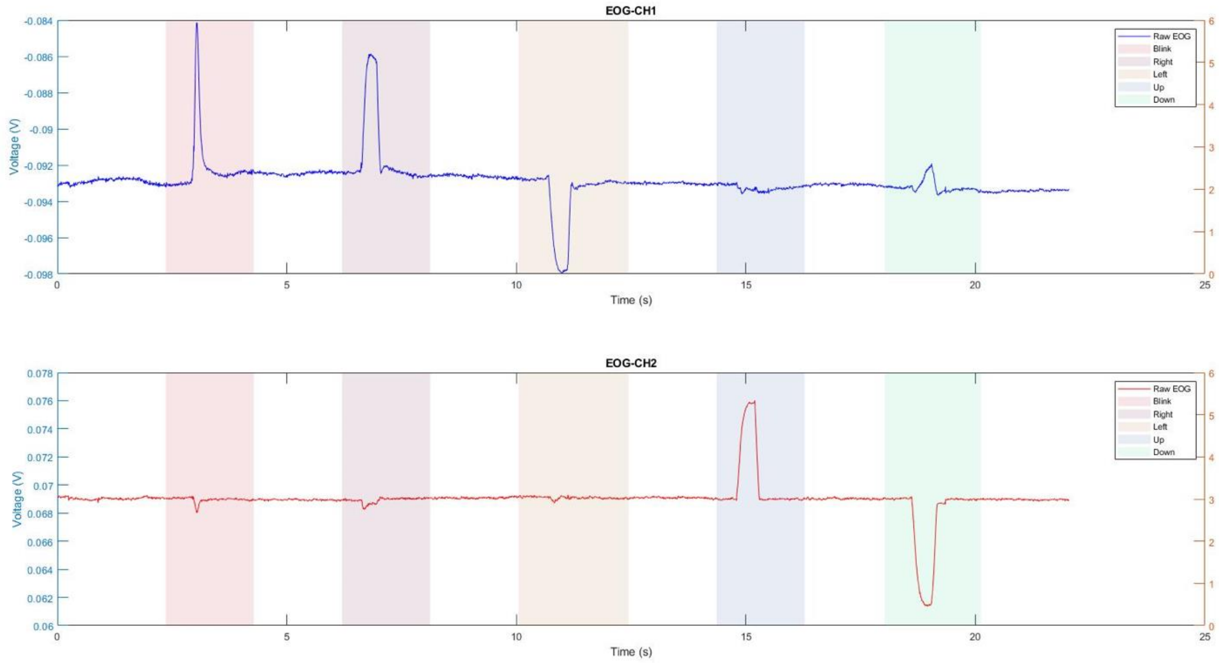

Figure S12. Raw EOG 2 channel data and classified result.

Table S1. The elastic moduli and Poisson's ratios of the studied materials

Material	Elastic modulus	Poisson's Ratio
Ecoflex	0.006 MPa	0.49
PI	2.5 GPa	0.49
Au	78 GPa	0.44

Table S2. List of functional chips used in the flexible wireless circuit.

Component	Description	Value	Part number
U1	3.3 voltage regulator	N/A	TPS63001
U2	Analog front-end	N/A	ADS1292
U3	Bluetooth PSoC	N/A	NRF52832-QFAA-R
U4	Motion sensor	N/A	MPU-9250
U5	Current limit active-low load switch	N/A	TPS22941
L1	0402 inductor	2.2 μH	N/A
L3	0402 inductor	15 nH	N/A
L4	0603 inductor	10 μH	N/A
L5	0402 inductor	10 nH	N/A
L6	0402 inductor	2.7 nH	N/A
C1, C10, C14, C15	0402 ceramic capacitor	10 μF	N/A
C2	0402 ceramic capacitor	22 μF	N/A
C3, C5	0402 ceramic capacitor	4.7 nF	N/A
C4	0402 ceramic capacitor	1.0 nF	N/A
C6, C7, C12, C22 C26, C33 C35, C36	0402 ceramic capacitor	0.1 μF	N/A
C8, C11, C13	0402 ceramic capacitor	1.0 μF	N/A
C9	0402 tantalum capacitor	1.0 μF	N/A
C21	0402 ceramic capacitor	4.7 μF	N/A
C23	0603 ceramic capacitor	10 μF	N/A
C24, C25 C28, C29	0402 ceramic capacitor	12 pF	N/A
C31	0402 capacitor	0.4 pF	N/A
R1, R2 R3, R4	0402 resistor	30 kΩ	N/A
R5, R6 R7, R8	0402 resistor	1 MΩ	N/A
R11, R12	0402 resistor	10 kΩ	N/A
A1	2.45 GHz RF chip antenna	N/A	2450AT18A100
F1	2.45 GHz low pass filter	N/A	2450FM07A0029
X1	32 MHz crystal	N/A	ECS-320-8-37CKM
X2	32.768 kHz crystal	N/A	ECS-.327-9-12-TR

Video S1. Real-time, continuous monitoring of EOG with a wearable device

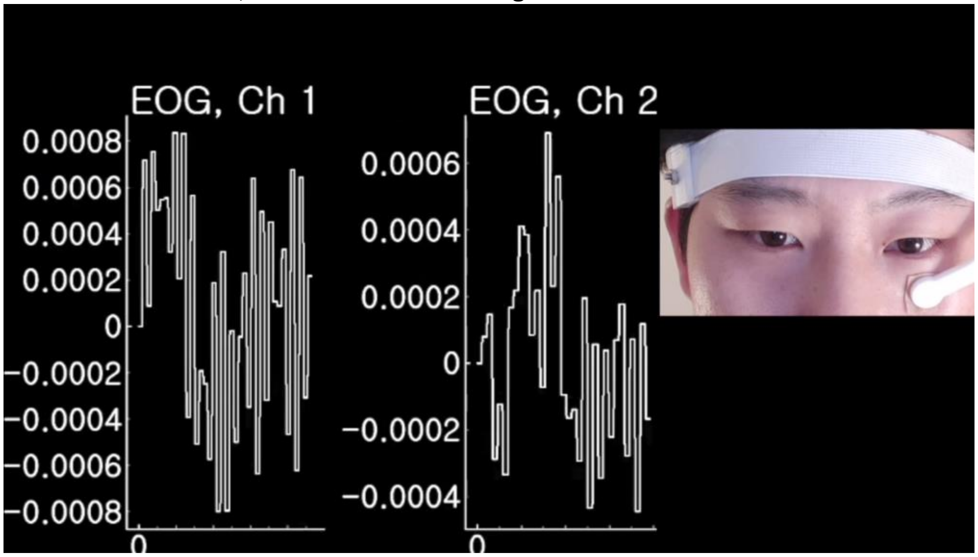

Video S2. Demonstration of wireless real-time control of a mini-drone car with the wearable device

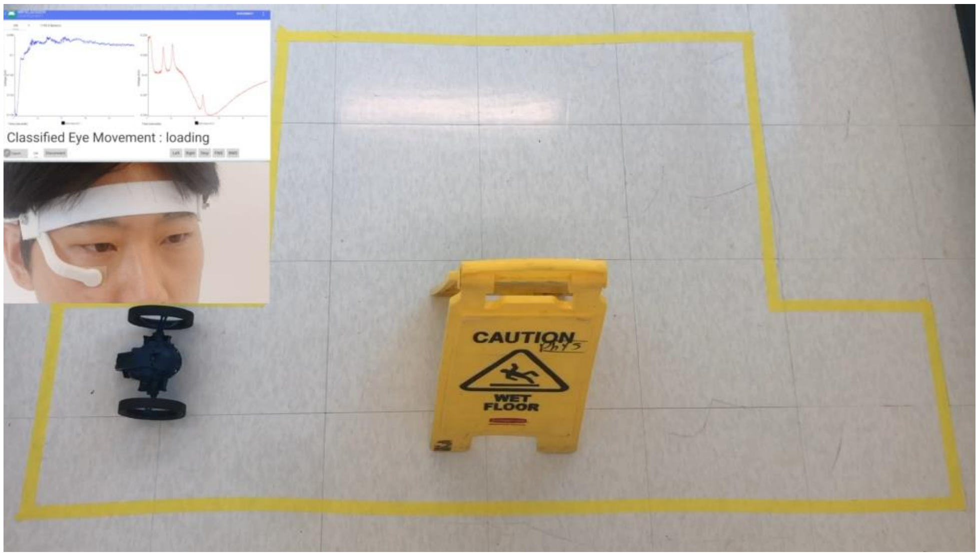

Supplement: Supplementary file 1 — el2c01436_si_001.pdf [file el2c01436_si_001.pdf]
